# Two-Tongued Cross-Finger Filet Flap Distal Digit Reconstructions Using Spare Parts After Multiple Distal Digital Amputations

**Published:** 2020-05-31

**Authors:** Ethan Song, Adrian Moy, Robert Teixeira, Sean J. Wallace, Yee Cheng Low

**Affiliations:** ^a^Morsani College of Medicine, University of South Florida, Tampa; ^b^Division of Plastic & Reconstructive Surgery, Department of Surgery, Lehigh Valley Health Network, Allentown, Pa

**Keywords:** digital amputation, cross finger flap, filet flap, mallet finger deformity, spare parts

## CASE DESCRIPTION

A 47-year-old right-hand-dominant male mechanic presented with a mutilating left-hand injury consisting of a small finger amputation distal to the level of the distal interphalangeal (DIP) joint with exposed bone, a complex near-complete amputation of the ring finger at the proximal interphalangeal (PIP) joint with a pulverized and missing middle phalanx head, and a dorsal long finger laceration resulting in a terminal extensor tendon laceration and an exposed DIP joint.

Soft-tissue coverage utilizing an improvised 2-tongued cross-finger flap was performed utilizing the proximally based spare parts secondary to the trauma. This was achieved by removing the nonreplantable and nearly amputated distal phalanx of the ring finger and dividing the ring finger soft-tissue longitudinally, thus creating 2 flaps. The radial flap was secured to the small finger amputation stump, and the ulnar flap was used to resurface the ring finger amputation stump site. Finally, the long finger DIP joint was washed out, the capsule closed, the terminal extensor tendon laceration repaired primarily, and a flexion-blocking splint placed. Three weeks later, at cross-finger flap division, he was noted to have a mallet deformity, so the long finger was percutaneously fixated into extension with a Kirschner wire. Postoperatively, he went on to have excellent clinical and functional outcomes without evidence of neuroma, cold insensitivity, or significant limitations in active or passive range of motion. He has since returned to work.

## QUESTIONS

1. How are fingertip amputations described and evaluated?
2. What are common methods of reconstruction of fingertip injuries?
3. How are mallet deformities classified?
4. How are mallet deformities treated?

## DISCUSSION

**Figure 1:**
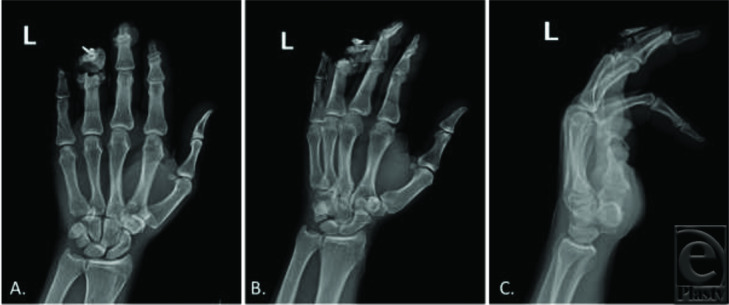
Three-view preoperative radiographs: (*a*) Posteroanterior, (*b*) oblique, and (*c*) lateral, demonstrating a left-hand small finger amputation distal to the level of the DIP joint, a near amputation and comminuted ring finger middle phalanx head fracture, and a long finger fracture-dislocation involving the DIP joint and distal phalanx. DIP indicates distal interphalangeal.

**Figure 2:**
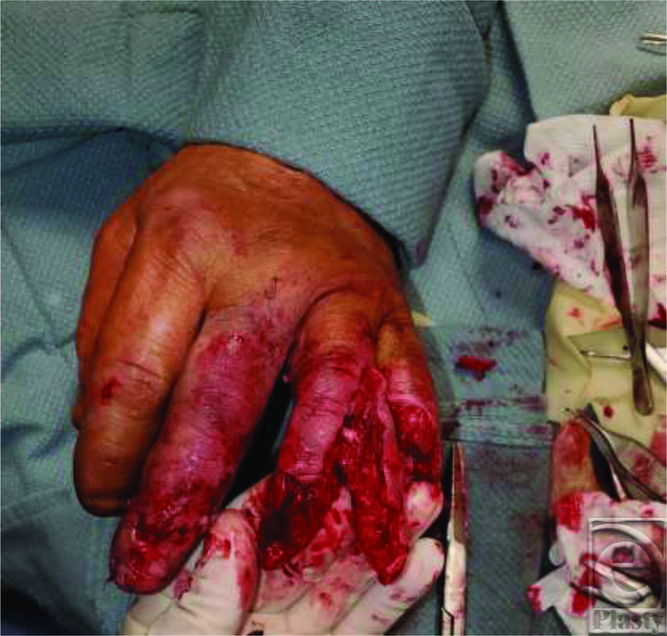
Clinical depiction of soft-tissue injury revealing small fingertip amputation, the ring finger after removing the mutilated distal phalanx and longitudinally splitting the soft-tissue spare parts into a 2-tongued filet flap, and repair of the long finger terminal extensor tendon and soft-tissue laceration.

Fingertip amputations are a common injury that can lead to significant functional consequences. Evaluation of fingertip amputations includes a thorough history gathering of age, gender, hand dominance, occupation, mechanism of injury, and existing comorbid illness. Along with a 3-view radiograph, the injured digit(s) should be examined for size, sensation, location, level of amputation, complete versus incomplete amputation, and geometry of injury. If available, the amputated segment should be examined in a similar fashion. Upon evaluation, our patient was diagnosed with a small fingertip amputation distal to the DIP joint, near amputation of the ring finger at the PIP joint with a pulverized middle phalanx head, and a fracture-dislocation with terminal extensor tendon laceration of the long finger at the DIP joint (Figs 1 and 2).

There are multiple classification schemes to further describe and facilitate evaluation of fingertip injuries. Within the Fassler^1^ classification scheme, the geometry of injury can be divided into volar oblique with or without exposed bone, transverse, and dorsal oblique. Vascular supply is further defined in the classification by Tamai, which identifies 5 zones, with zone areas increasing as the area becomes more proximal. A more recent PNB classification system was introduced by Evans and Bernadis based on 3 components of pulp (P), nail (N), and bone (B). While the PNB classification provides granularity by assigning digit grades to these components, there have been issues with interobserver reliability.^2^

**Figure 3:**
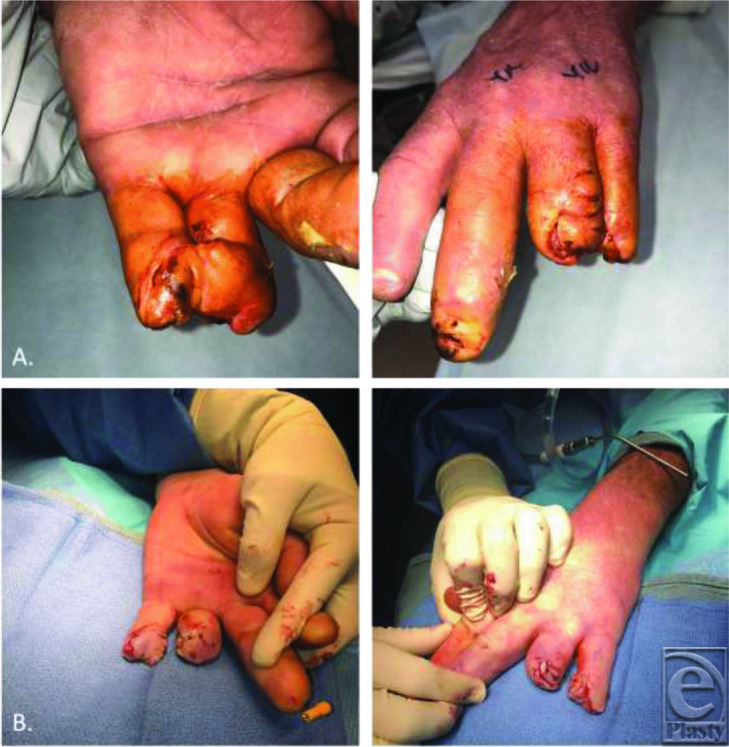
Dorsal and volar views of (*a*) the 2-tongued cross-finger filet flap at 3 weeks just prior to pedicle division and (*b*) after cross-finger flap division and inset, as well as percutaneous skeletal fixation of the long finger mallet deformity with a Kirschner wire.

The goals of surgical correction of amputations include preserving length, coverage and sensation, minimizing postoperative morbidity, and providing an acceptable cosmetic appearance. Strategies follow the reconstructive ladder and can vary depending on the specific presentation of injury. This includes healing by secondary intention, skin grafting, homodigital and heterodigital flap reconstruction, and microsurgical replantation. Given the nature of our patient's mutilating digital injury involving the ring and small fingers, a modified cross-finger flap utilizing spare parts was determined to be the best option. Classically, cross-finger flaps can cover larger defects than homodigital flaps and are useful for large volar oblique injuries. First described in 1950 by Gurdin and Pangman,^3^ cross-finger flaps are often used for ring and small finger reconstruction. However, postoperative sensibility can be poor, as a study of 54 patients revealed 0% tactile gnosis and 53% cold insensitivity.^4^ Improvisation with a 2-tongued cross-finger filet flap allowed for coverage of the ring finger stump, as well as resurfacing of the small finger amputation site to maintain digital length (Fig 3).

Mallet finger is another common injury of the fingertip, often secondary to trauma such as forceful flexion against an extended digit or a laceration about the DIP joint.^5^ There may be extensor tendon damage or a distal phalanx avulsion-fracture with loss of active extension at the DIP joint. In this case, the trauma resulted in a terminal extensor tendon laceration of the long finger, causing a mallet deformity. The Doyle system is used to classify mallet fingers as follows: type 1, closed with or without avulsion fracture; type 2, open with tendon laceration; type 3, open with tendon substance and soft-tissue loss; and type 4, mallet fracture.^6^ Type 4 is subdivided into transphyseal fractures (type 4A), hyperflexion with involvement of 20% to 50% of the articular surface (type 4B), and hyperextension involving more than 50% of the articular surface (type 4C).

**Figure 4:**
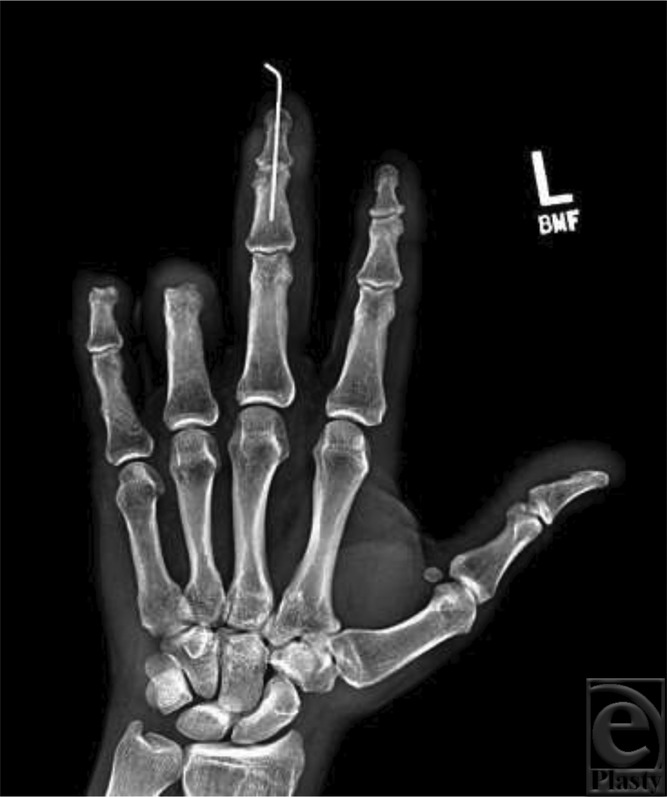
Postoperative radiograph demonstrating soft-tissue coverage of the amputation stumps of the small fingertip and the ring finger at the PIP joint. Also shown is skeletal fixation of the long finger DIP joint for treatment of the mallet deformity. PIP indicates proximal interphalangeal; DIP, distal interphalangeal.

Treatment of mallet deformities includes nonoperative versus surgical management. First-line therapy is typically nonoperative, consisting of immobilization of the DIP joint in full extension for 6 weeks, followed by 6 weeks of nighttime splinting. Nonoperative management has been effective for both uncomplicated and complicated mallet injuries. Surgery is indicated for complex fractures, distal phalanx fractures involving more than 30% of the articular surface, or in volar subluxation of the distal phalanx.^7^ In our patient, percutaneous skeletal fixation was performed because of recurrent mallet deformity at 3 weeks, along with his desire to return to work (Fig 4). Open reduction and fixation (ORIF) techniques can be considered and have similar efficacy but may have a higher complication rate. As in this case, ORIF may be accomplished with a Kirschner wire or alternatively with a hook plate and/or screws. In cases where deformity or functional impairment persists following initial management, arthrodesis may be performed.^5^^,^^7^^,^^8^

## SUMMARY

We presented a case of a patient who suffered complex and mutilating fingertip injuries following trauma that were successfully reconstructed with an improvised spare parts cross-finger filet flap. The longitudinal ring finger laceration presented a unique opportunity for creative spontaneity of the cross-finger flap by creating a 2-tongued filet flap from spare parts to provide not only soft-tissue coverage of the ring finger but also preservation of length of the small fingertip amputation stump.
